# Prioritizing Populations for Conservation Using Phylogenetic Networks

**DOI:** 10.1371/journal.pone.0088945

**Published:** 2014-02-28

**Authors:** Logan Volkmann, Iain Martyn, Vincent Moulton, Andreas Spillner, Arne O. Mooers

**Affiliations:** 1 Department of Biological Sciences, Simon Fraser University, Burnaby, British Columbia, Canada; 2 Interdisciplinary Research in the Mathematical and Computational Sciences (IRMACS) Centre, Simon Fraser University, Burnaby, British Columbia, Canada; 3 School of Computing Sciences, University of East Anglia, Norwich, United Kingdom; 4 Department of Mathematics and Computer Science, University of Greifswald, Greifswald, Germany; Natural History Museum of Denmark, Denmark

## Abstract

In the face of inevitable future losses to biodiversity, ranking species by conservation priority seems more than prudent. Setting conservation priorities within species (i.e., at the population level) may be critical as species ranges become fragmented and connectivity declines. However, existing approaches to prioritization (e.g., scoring organisms by their expected genetic contribution) are based on phylogenetic trees, which may be poor representations of differentiation below the species level. In this paper we extend evolutionary isolation indices used in conservation planning from phylogenetic trees to phylogenetic networks. Such networks better represent population differentiation, and our extension allows populations to be ranked in order of their expected contribution to the set. We illustrate the approach using data from two imperiled species: the spotted owl *Strix occidentalis* in North America and the mountain pygmy-possum *Burramys parvus* in Australia. Using previously published mitochondrial and microsatellite data, we construct phylogenetic networks and score each population by its relative genetic distinctiveness. In both cases, our phylogenetic networks capture the geographic structure of each species: geographically peripheral populations harbor less-redundant genetic information, increasing their conservation rankings. We note that our approach can be used with all conservation-relevant distances (e.g., those based on whole-genome, ecological, or adaptive variation) and suggest it be added to the assortment of tools available to wildlife managers for allocating effort among threatened populations.

## Introduction

Extinctions due to human impacts are now unavoidable: even optimistic scenarios predict significant changes in biodiversity by the year 2100 [1], [2], with most extinction starting with the loss of isolated populations [3], [4].

**Figure 1:**
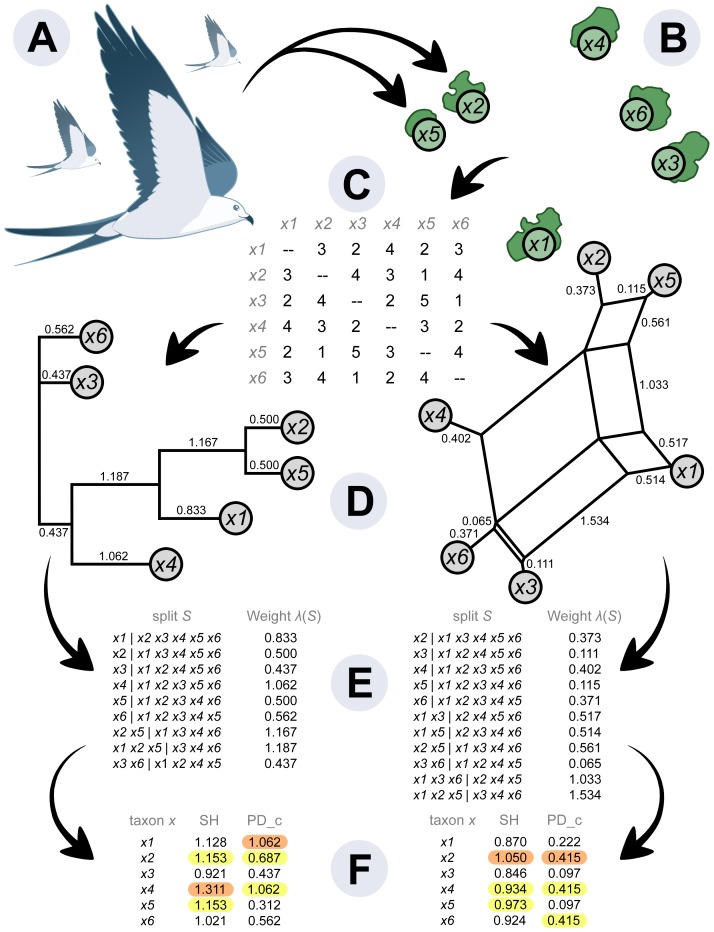
Using pairwise distances to rank species or populations. Consider a hypothetical group of taxa (A)—a set of closely-related species or populations of a single species—that is distributed across several islands in an archipelago (B). Differences among the taxa, labeled *x1* through *x6*, can be organized into a pairwise distance matrix (C). We can represent this matrix either as a phylogenetic tree or as a phylogenetic network (D), where a set of weighted *splits* describes the relationships among the taxa (E). Altogether, these splits represent the group's phylogenetic diversity (PD). By selecting subsets of splits that exclude a given taxon, we can calculate each taxon's contribution to the total PD of the tree or network (F). The *Shapley metric* (SH) and *expected PD complementarity* (PD_c) are different approaches for ranking taxa based on split data. Note that the highest-scoring taxa (highlighted values) can differ considerably depending on the type of metric used and whether the splits come from a tree or network. We discuss the reasons for these differences and methods for ranking taxa in Section (*ii*) of the main text.

One prime conservation goal is to preserve genetic variation [5], [6], both as a representation of past evolution and raw material for future evolution [7] and, potentially, as a surrogate for improved ecosystem function [8]. However, not all genetic lineages are equally important, with more isolated lineages warranting additional interest because of their expected contribution to total variation [5], [8], [9]. Indices of evolutionary isolation have been developed to rank species on a phylogenetic tree based on unique and shared evolutionary history (*e.g.*, [10]–[13]). These metrics use rooted phylogenetic trees with edge lengths as input (Figure 1), and rank tips with less shared history as requiring more urgent conservation attention. For example, the Zoological Society of London has made this approach operational in their “Edge of Existence” programme (www.edgeofexistence.org). In the United States, taxonomic distinctiveness is one of several explicit criteria for prioritizing conservation attention [14]. The extension to populations within species would seem to be straightforward.

Below the species level, Ryder [15] advocated the use of *evolutionarily significant units* (ESUs) to identify populations with genetic variation in need of long-term conservation; this was expanded by Moritz [16] and Waples [17] to the concepts of *management units* (MUs) and *distinct population segments* (DPS), respectively, for species that had undergone more recent range fragmentation. All these population-based approaches have enjoyed wide usage in population genetic studies (*e.g.*, [18]–[21]), and are the basis for identifying populations worthy of protection in law. Importantly, ESUs assume that the relationships among populations can be represented by a bifurcating tree. However, bifurcating trees often fail to capture the relationships among populations [22]. DPSs and MUs can deal with populations that have more complex interrelationships (Figure 1), but neither of these designations is designed to prioritize among populations. This would seem a major shortcoming if populations do need to be prioritized for conservation attention.

Previous authors have shown that the logic of measuring and maximizing phylogenetic diversity [6], which forms the basis for tree-based prioritization schemes, can be generalized to phylogenetic networks [23]–[25]. Here we show that the prioritization approaches for trees can also be adapted for populations within species by extending evolutionary isolation indices from trees to networks. We develop efficient algorithms to compute these indices for NeighborNet networks [26], [27], and illustrate their use with heuristic data from two imperiled species, the spotted owl (*Strix occidentalis* Xantus de Vesey 1860) and the mountain pygmy-possum (*Burramys parvus* Broom 1896). The new approach to assessing population differentiation might be of immediate practical use to those tasked with managing discrete populations of a threatened species, and may allow for new policy associated with conservation triage [28].

## Methods

We present our approach for prioritizing populations in three steps. First, we briefly review the various approaches for measuring diversity and evolutionary isolation on bifurcating trees of taxa. We then review the properties of NeighborNet networks as a representation of pairwise evolutionary distances and describe how to prioritize taxa by their expected contribution to biodiversity. In File S1, we outline efficient algorithms for estimating evolutionary isolation on NeighborNet networks. Finally, we illustrate the new method of population prioritization using two small published datasets.

### (i) Diversity measures on trees and networks

The concept of evolutionary isolation can be understood in terms of a species' biological distinctiveness, which we might measure by comparing its adaptive or non-adaptive traits to those of related species. More generally, our goal is to measure a taxon's contribution to the current and/or future “diversity” in a set of taxa. Several different approaches for quantifying such diversity have been proposed. One of the earliest, described by Weitzman [23], is *expected diversity*. Rather than score taxa individually, this approach seeks to identify the set of taxa that will retain the most diversity on a future tree, given some measure of diversity and a probability of persistence for each potential combination of taxa. Although Weitzman's original diversity metric was rather general, he did consider an example of biological character-state differences that could be represented on a phylogenetic tree.

On such a tree, every taxon contributes an amount of unique evolutionary information denoted by the length of the branch (or *edge*) linking it to all other taxa (Figure 1) [6], [23]. This length may be calibrated in units of time (*e.g.*, millions of years) or in raw or inferred genetic distances. Looking specifically at biological systems, Witting and Loeschcke [29] and Faith and Walker [30] combined Weitzman's [23] expected diversity framework with Faith's [6] concept of *phylogenetic diversity* (PD), the latter which specifically calculates the sum of all branch lengths on a tree (see next section). Like Weitzman [23], this *expected PD* approach can be used to identify a set of taxa that maximizes the amount of total tree length retained, given a set of extinction probabilities for the tips.

The related *k of n problem* [6] seeks to identify the most diverse subset of *k* taxa (*i.e.*, the one that maximizes PD) on a tree of size 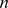. Faith [31] and Weitzman [32] explored the special case where 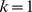, which Faith [33] refers to as the *PD complementarity* of a given taxon.

An independently-derived approach based on Game Theory ([10], first published 2005) explicitly considers the individual contribution of each taxon to future diversity. Like Weitzman's [23] expected diversity framework, all possible subsets of taxa on a tree may persist. By calculating the amount of unique information each taxon contributes to future subsets (*i.e.*, the average length of the edge linking the taxon to all possible future trees), one can rank taxa in order of their relative impact on future diversity. This *Shapley metric* (SH) is almost identical to the ad-hoc *evolutionary distinctness* (ED) metric used by the Zoological Society of London in their Edge of Existence programme (www.edgeofexistence.org). The major difference between the two is that the ED metric is explicitly measured on a rooted tree, as opposed to the more general undirected graph that SH takes as input [34].

The Shapley metric was further refined by Steel *et al*. [35] and named HED (for *heightened evolutionary distinctiveness*). HED is the expected contribution of a given taxon to future subsets of taxa where the subsets are weighted by their probability of persistence. In this case, the focal taxon is assumed to persist (*i.e.*, its probability of extinction does not affect its HED score). On trees, HED is formally equivalent to a form of PD complementarity where the contribution of a taxon is measured with respect to all possible subsets, each weighted by their probability of persistence [33]. Weitzman [32] also arrived at this formulation ten years earlier, which he termed the “distinctiveness” of a taxon, in the context of his “Noah's Ark Problem” of biodiversity preservation. Using Faith's [33] terminology, HED, which combines the concepts of expected PD with PD complementarity, might be considered *expected PD complementarity*.

As a final antecedent, Minh *et al*. [24], [25], [36] extended PD to phylogenetic networks and presented algorithms for solving the *k* of *n* problem to maximize diversity for a given subset size. They referred to this metric as *split diversity* (SD).

In this context it should be possible to measure the PD contribution of individual taxa on a phylogenetic network. Critically for our purposes, the two metrics we use here (SH and HED) do not require a rooted phylogenetic tree, and so can be adapted to networks in the same way that PD indices can [24], [25], [27], [36]. SH and HED are formally defined in File S1 and discussed further below. In short, if we do not have probabilities of extinction for taxa, we assume all future subsets of taxa are equally likely, and calculate SH. If we can estimate (even broadly) the probabilities of persistence of all taxa, we can weight future subsets by their probability, and use HED.

### (ii) Interpreting phylogenetic networks, Shapley values, and HED

NeighborNet [26] is a method that permits the representation of pairwise distances between taxa in the form of a network. An important property of NeighborNet networks is that they permit the representation of relationships among the underlying taxa that cannot be depicted on any phylogenetic tree. For example, to the extent that populations exchange migrants, the between-population genetic distance data (*F*_ST_) may yield many alternative trees, none of which accurately reflect the actual relationships among these populations (*e.g.*, [37]). The NeighborNet framework, by contrast, accommodates for such phylogenetic uncertainty and will always yield a single network with positive edge lengths, permitting calculation of SH and HED. If a pairwise distance matrix is tree-like (*i.e.*, yields only one possible phylogeny) the resulting NeighborNet output will resemble a phylogenetic tree. Where there is no tree-like history, a network representation should be more informative. Indeed, for many distance matrices (including Example A below, results not shown), the assumptions necessary to produce a tree are not met, and a neighbor-joining tree, for example, produces negative edge lengths. Here, a network representation would definitely be preferred [26].

An example of a very simple matrix of pairwise distances and the resulting network is depicted in Figure 1. Each edge or set of parallel edges in the network corresponds to a partition of the underlying set of taxa into two non-overlapping subsets, called a *split* (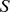). The *edge length* reflects the *weight* of the split (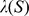)—in other words, a component of the pairwise distance (*F*_ST_, for example) separating any two taxa. Thus, just as a phylogenetic tree represents a collection of weighted splits (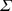) [38], where each branch of the tree denotes a split, a NeighborNet network represents a weighted collection of splits of the underlying set of taxa. As Figure 1 illustrates, the distance between two tips on a network (*i.e.*, the shortest path between two taxa) represents the observed distance in the distance matrix.

Whether represented on a tree or a network, every split system contains information on the overall diversity of its constituent taxa [5], [23]. The conservation planning metric *phylogenetic diversity* (PD) [6] can be calculated for split systems as
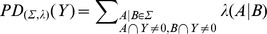



where 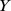 is a subset of taxa on the tree or network and 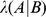 is the weight of the split between two non-overlapping groups 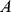 and 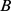 of taxa. Note that the overall PD for both trees [6], [39] and networks [24], [25], [36] is simply the sum of all split weights (Figure 1).

A very simple approach for measuring an individual taxon's PD contribution, illustrated in Figure 1, is to consider the change in PD when this taxon is removed from the tree or network [40]. This *PD complementarity* (PD_c) metric can be expressed as
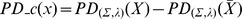



where 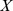 is the set of all taxa in the tree or network and 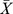 is the subset where a given taxon 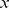 has been removed from the underlying distance matrix.

We can also extend the metrics SH and HED from trees to NeighborNet networks using similar ideas for extending PD calculations from trees (*e.g.*, [6], [27], [29]) to networks (*e.g.*, [24], [25], ). On a tree, the Shapley value (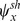) for taxon 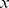 can be defined as the mean split weight of the set of splits defining 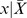, where 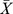 represents all unique possible subsets of the taxon set 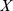 that do not contain 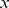. Importantly, Haake *et al*. [10] present a formal proof that the Shapley value for 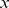 can also be calculated as a weighted sum of all the edge lengths on a tree, with the weights determined by the sizes of the sets containing 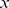. This can be presented compactly using split notation as
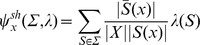



where 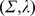 is the set of splits defined by the network and their weights, 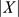 is the total number of taxa, 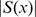 is the size of a split set containing the taxon 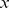, 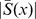 is the size of the complementary set that does not contain 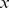, and 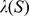 (following the notation from Minh *et al*. [24], [25]) is the split weight, equal to the edge length separating 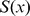 from 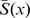. To calculate the Shapley value for taxon 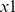 in the network in Figure 1, we take the first split 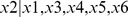 to be composed of 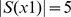 and 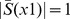 and 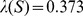, the second split 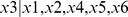 to be composed of 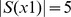 and 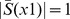 and 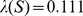 and so on. With a taxon set containing six elements, 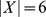 and the Shapley value for taxon 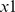 is 0.870 (Figure 1).

As with a phylogenetic tree, the sum of Shapley values will always equal the sum of all parallel split weights in the network. Because the shape of a network reflects the relative distances among its taxa, we should expect outlying taxa (*i.e.*, those connected to the rest of the network by long edges, like taxon 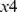) to show higher values for 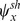. Thus, the Shapley values calculated for a network can reflect the relative degree of isolation of each taxon based on molecular, morphological, or any other relevant distance measure.

Though conceptually similar, the calculation of HED (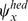) is somewhat more complex, as it accounts for differences in the probability of extinction 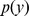 for each taxon: 
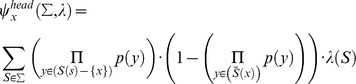



Here, the first product operator considers 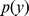 for every taxon in 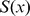 but excludes 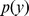 for taxon 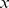 itself [27], [35]. The second product operator considers 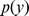 for every taxon in 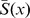. Unlike SH, the sum of HED scores will not equal the sum of split weights in the split system. We also note that 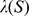 will influence HED more strongly than 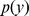 for outlying taxa. Thus, the ranking order for highly isolated populations should be similar for SH and HED, regardless of which populations have a higher extinction probability.

A more detailed mathematical treatment of the SH and HED metrics and efficient algorithms for their computation are given in File S1. For the datasets in this paper, we used the implementation of NeighborNet in the SplitsTree software package [41] to compute networks. For a given matrix of pairwise distances, this yields the network together with the corresponding collection of weighted splits. We also developed custom R scripts (available in File S1) [42] to compute SH and HED on the outputs from SplitsTree.

### (iii) Application

We present SH and HED ranking for two datasets based on putatively neutral genetic markers. In the first example (*A*), the size of each population (and hence the probability of extinction for each population) is not known. In the second example (*B*), population sizes are known, allowing us to estimate separate probabilities of extinction for each population.

We selected our two examples based on the following criteria: (1) The species as a whole is of conservation interest (*i.e.*, vulnerable, endangered, or critically endangered), (2) its distribution is fragmented (*i.e.*, we can define multiple populations), (3) sampling efforts have covered its entire range, and (4) genetic analyses have been published or the raw sequence data made publicly available.

Readers should note that the primary goals of this article are to introduce and illustrate our network ranking approach, not to advocate new management decisions for the taxa described below.

#### Example A

**Figure 2:**
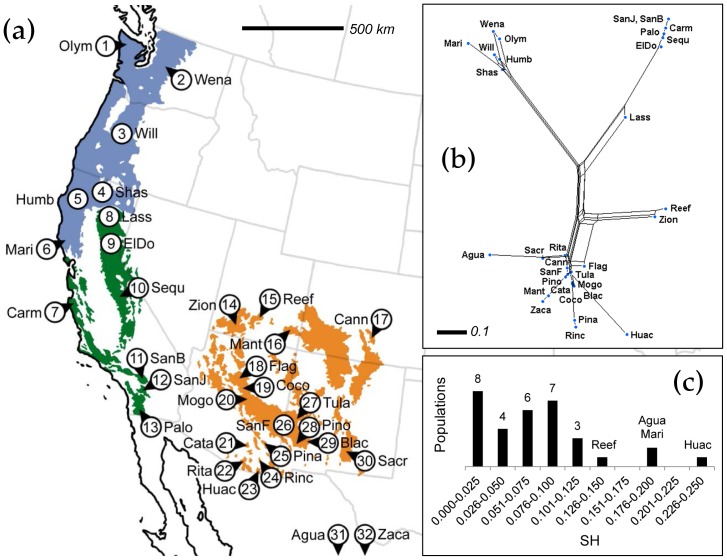
Conservation prioritization of spotted owl (*Strix occidentalis*) populations. (a) Distribution of spotted owls in the United States and the populations sampled by Barrowclough *et al.* [48], [51]. Shaded areas denote suitable habitat based on forest cover data [73]. Colors denote the subspecies *S. o. caurina* (blue), *S. o. occidentalis* (green), and *S. o. lucida* (orange). Populations 31 and 32 represent the *S. o. juanaphillipsae* subspecies in Mexico (range not shown). (b) NeighborNet of sampled populations based on mtDNA differentiation (pairwise Φ_ST_ values). (c) Histogram of SH values, highlighting the populations with the highest scores. See Table 1 for an explanation of abbreviations used.

Spotted owls (*Strix occidentalis*) are distributed throughout late-succession conifer forests in western North America [43]. Four subspecies are currently recognized (Figure 2a): *S. o. caurina* from southern British Columbia to northwest California, *S. o. occidentalis* in California and Nevada, *S. o. lucida* in Utah, Colorado, Arizona, New Mexico, and northern Mexico, and *S. o. juanaphillipsae* in central Mexico [44], [45]. Populations in the United States continue to decline due largely to poor timber harvesting practices, but also as a result of climate change and the westward expansion of barred owls (*S. varia* Barton 1799) [46]. *S. o. caurina* (the northern spotted owl) and *S. o. lucida* (the Mexican spotted owl) are threatened subspecies under the United States' Endangered Species Act, and *S. o. occidentalis* (the California spotted owl) is a subspecies of special concern in the state of California [47]. Spotted owls in the American Southwest “sky islands” (mostly *S. o. lucida*) are particularly fragmented and perhaps most suitable for population-level conservation [48]. Although genetic data for the Mexican subspecies remain poor, we can construct a reasonably complete representative phylogenetic network for subspecies in the United States.

Spotted owl mitochondrial sequences were obtained from Genbank (accession numbers AY833608–AY833644, AY836774–AY836776, DQ230843–DQ230888) and aligned in Mega v. 5 [49] using MUSCLE [50]. These sequences comprise about 1105 bp of the control (D-loop) region and represent 86 haplotypes from 32 populations in the United States and Mexico (Figure 2b; Table 1) [48], [51]. We ran a standard analysis of molecular variance (AMOVA) [52] on all 298 aligned sequences in Arlequin v. 3.5 [53] using the Kimura 2-Parameter model [54] to compute distances among haplotypes (Φ_ST_). This procedure generated a pairwise differentiation matrix for the 32 populations (Table S1). A NeighborNet based on this matrix (Figure 2b) [26] was then constructed in SplitsTree v. 4.11 [41] under default assumptions. Negative Φ_ST_ values were treated as being equal to zero. Because the size of each population is not known, for the purposes of illustration, we gave each population an extinction probability 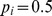 when calculating HED—an approach similar to the “PD50” metric used by FISHBASE (www.fishbase.org) [55].

**Table 1:** Spotted owl populations sampled by Barrowclough *et al*. [48], [51] and ranked by Shapley value (SH) and heightened evolutionary distinctiveness (HED).

Pop.	Code	Subspecies	State	Sampling Locality	*n* ind.	*n* hap.	SH	HED
1	Huac	*lucida*	AZ	Huachuca Mountains	5	2	0.242	7.431E-03
2	Agua	*juanaphillipsae*	—	Aguascalientes, Sierra Fria, Mexico	1	1	0.191	3.983E-03
3	Mari	*caurina*	CA	Marin County	8	3	0.177	4.067E-03
4	Reef	*lucida*	UT	Capitol Reef National Park	9	4	0.133	3.683E-03
5	Wena	*caurina*	WA	Cascade Range	10	8	0.111	1.777E-03
6	Olym	*caurina*	WA	Olympic Peninsula	10	4	0.106	1.729E-03
7	Zion	*lucida*	UT	Zion National Park	7	4	0.105	2.851E-03
8	SanB	*occidentalis*	CA	San Bernardino Mountains	15	1	0.091	9.418E-04
9	SanJ	*occidentalis*	CA	Mount San Jacinto	15	1	0.091	9.418E-04
10	Rinc	*lucida*	AZ	Rincon Mountains	8	4	0.089	2.542E-03
11	Will	*caurina*	OR	Willamette National Forest	15	8	0.081	7.621E-04
12	Carm	*occidentalis*	CA	Carmel Valley	10	1	0.079	5.187E-04
13	ElDo	*occidentalis*	CA	El Dorado National Forest	15	4	0.077	6.214E-04
14	Zaca	*juanaphillipsae*	—	Zacatecas, Sierra de Urica, Mexico	1	1	0.076	2.079E-03
15	Palo	*occidentalis*	CA	Mount Palomar	8	1	0.074	3.994E-04
17	Humb	*caurina*	CA	Humboldt and Siskiyou Counties	30	11	0.072	4.528E-04
16	Sequ	*occidentalis*	CA	Sierra National Forest	15	6	0.071	3.422E-04
18	Pina	*lucida*	AZ	Pinaleno Mountains Graham County	4	2	0.071	1.605E-03
19	Shas	*caurina*	CA	Klamath and Shasta National Forests	16	8	0.067	3.638E-04
20	Mant	*lucida*	UT	Manti-La Sal National Forest	2	2	0.054	1.276E-03
21	Sacr	*lucida*	NM	Sacramento Mountains	8	6	0.047	1.284E-03
22	Lass	*occidentalis*	CA	Lassen National Forest	11	6	0.041	1.006E-04
23	Flag	*lucida*	AZ	San Fransisco Peaks	4	4	0.027	2.144E-04
24	Blac	*lucida*	NM	Black Range	8	6	0.026	3.461E-04
25	Coco	*lucida*	AZ	Coconino Plateau	15	9	0.022	1.039E-04
26	Cata	*lucida*	AZ	Santa Catalina Mountains	5	3	0.021	6.359E-05
27	Pino	*lucida*	NM	Pinos Altos Mountains	5	4	0.020	2.832E-05
28	SanF	*lucida*	NM	San Fransisco Mountains	7	4	0.017	1.020E-06
29	Cann	*lucida*	CO	Near Canon City	4	4	0.017	8.505E-07
30	Rita	*lucida*	AZ	Santa Rita Mountains	4	4	0.017	1.374E-05
31	Mogo	*lucida*	AZ	Mogollon Mesa	8	6	0.017	8.578E-07
32	Tula	*lucida*	NM	Tularosa Mountains	15	12	0.017	2.034E-06

Number of individuals (*n* ind.), number of haplotypes (*n* hap.), SH, and HED scores from the present study are reported.

#### Example B

**Figure 3:**
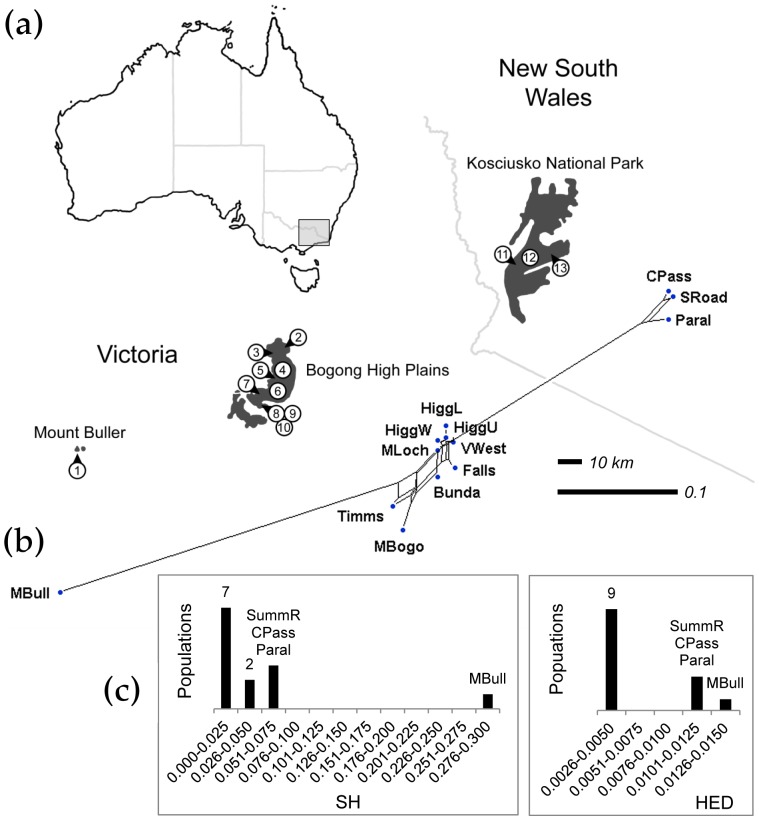
Conservation prioritization of mountain pygmy-possum (*Burramys parvus*) populations. (a) Distribution of mountain pygmy-possums in Australia (gray inset), showing populations sampled by Mitrovski *et al.* [58]. Shaded areas denote suitable habitat above 1,400 m. (b) NeighborNet of sampled populations based on microsatellite differentiation (pairwise *F*_ST_ values). (c) Histograms of SH and HED values, highlighting the populations with the highest scores. See Table 2 for an explanation of abbreviations used.

Mountain pygmy-possums (*Burramys parvus*) are alpine specialists restricted to three small regions of the Australian Alps (Figure 3a). The species depends on block streams and block fields found above 1,400 meters—habitats less than 10 km^2^ in total extent [56]. The areas where mountain pygmy-possums still occur are particularly sensitive to destruction and fragmentation. Surveys conducted in the 1990s estimated the adult population size to be 2,600 [57]. A decade later this number had decreased to below 2,000 [56], with signs of continued decline [58]. At present, the IUCN lists mountain pygmy-possums as critically endangered [59].

Because of its restricted distribution and high extinction risk, the species has been subject to extensive population genetic research [58], [60]–[62]. Unlike our example with spotted owls, direct estimates of population sizes are available, within-population sample sizes are uniformly large, and genetic data are available across the mountain pygmy-possums' entire range. This provides us with an opportunity to compare SH to HED and assess the effect of variable population sizes on conservation ranking.

We used a published matrix of genetic differentiation (*F*_ST_) based on data from 8 microsatellite loci [58] to construct a phylogenetic network for 13 mountain pygmy-possum populations (Figure 3b). Our methods for generating NeighborNet outputs, and for computing SH and HED, were the same as above.

We modeled the probabilities of extinction for individual populations (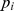) of a given size (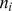) as a negative exponential 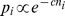



where the constant of proportionality 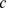 is 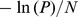, with 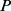 being the probability that the entire species goes extinct and 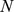 being the total census size of the species (the sum of 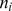). We used a conservative 100-year extinction probability for the entire species, 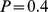, to derive HED (see [63]).

## Results

### Example A

As expected for a set of lineages with a recent history of gene flow, the network for spotted owls is quite non tree-like (Figure 2b). However, populations with the greatest degrees of genetic differentiation, relative to all other populations, occupy nodes subtending the longest edges. Populations at relatively isolated nodes, such as those from Mount San Jacinto and the Huachuca Mountains, share few mutations with neighboring populations and subsequently exhibit higher pairwise Φ_ST_ values (Table 2; Table S1). Conversely, the (uncorrected) pairwise Φ_ST_ values for closely-related populations are either negative (as great as -1) or close to zero, indicating higher levels of genetic differentiation within these populations than among them [52].

**Table 2:** Mountain pygmy-possum populations sampled by Mitrovski *et al*. [58] and ranked by Shapley value (SH) and heightened evolutionary distinctiveness (HED).

Pop.	Code	Region	Sampling Locality	*n* ind.	*n* all.	*r*	*N*	*p*i	SH	HED
1	MBull	Southern	Mount Buller	66	3.38	2.29	150	0.9072	0.292	1.497E-02
2	CPass	Northern	Charlottes Pass	44	6.00	5.21	45	0.9712	0.072	1.153E-02
3	Paral	Northern	Paralyser	40	6.63	5.77	22	0.9858	0.071	1.089E-02
4	SummR	Northern	Summit Road	43	6.13	5.16	25	0.9839	0.068	1.138E-02
5	MBogo	Central	Mount Bogong	42	6.50	5.77	100	0.9372	0.045	4.497E-03
6	Timms	Central	Timm Spur	120	6.88	5.32	120	0.9251	0.028	3.859E-03
7	Falls	Central	Falls Creek	35	6.63	5.73	30	0.9807	0.020	3.051E-03
8	HiggL	Central	Mount Higginbotham L	17	5.25	5.25	50	0.9681	0.019	2.850E-03
9	Bunda	Central	Bundara	78	7.25	5.45	120	0.9251	0.018	2.858E-03
10	HiggW	Central	Mount Higginbotham W	59	7.63	6.01	250	0.8502	0.013	2.659E-03
11	VWest	Central	Pretty Valley West	69	7.00	5.95	50	0.9681	0.010	2.541E-03
12	HiggU	Central	Mount Higginbotham U	56	7.25	5.76	50	0.9681	0.010	2.420E-03
13	Mloch	Central	Mount Loch	93	7.00	5.80	400	0.7714	0.010	2.646E-03

Number of individuals (*n* ind.), number of alleles (*n* all.), allelic richness (*r*), and adult population sizes (*N*) are reported from previously-published data. Probabilities of extinction (*p*i, with *P* = 0.4), SH, and HED scores from the present study are also shown.

We observe strong geographic structure across the United States consistent with current subspecific designations (Figure 2b). Populations of *S. o. lucida* exhibit a more star-like phylogenetic network that may reflect historical isolation in the “sky islands” of the American Southwest [48]. The intermediate position of the Lassen National Forest population, in contrast, may be due to its location near the point of contact between southern *S. o. caurina* and northern *S. o. occidentalis* [51].

The results of our SH and HED ranking are shown in Table 1. As expected, populations at relatively isolated tips score higher than those closer to the interior of the network (Figure 2b, c). The rankings are highly consistent between the two metrics (Spearman rank correlation  = 0.91), and the same populations receive top ranking for both SH and HED.

### Example B

As with spotted owls, the most genetically differentiated populations of mountain pygmy-possums, namely those in the northern and southern areas of their range, occupy nodes that are separated from most other populations by long edges (Figure 3b). Overall the structure of our network is in good agreement with the species' present distribution (Figure 3a). Given the habitat requirements and limited dispersal ability of mountain pygmy-possums, it is not likely that Mount Buller and Kosciusko National Park still exchange migrants with the Bogong High Plains [58]. In contrast, the close grouping of central populations in our phylogenetic network, and subsequently their low SH and HED, is consistent with a shared history and/or recent gene flow.

The ranking results are shown in Table 2. Again, the phylogenetic network for mountain pygmy-possums reflects geographic distribution. Although we did not make *a priori* group assignments based on sampling location, the 13 populations still partition into northern, central, and southern regions. Again, outlying populations on the network tend to receive higher SH and HED scores. Unsurprisingly, the small and isolated Mount Buller population consistently ranks highest. For HED, no bias towards small or large populations is apparent; populations with high extinction probabilities do not necessarily receive high scores [35]. Again, although ranking order changes slightly between SH and HED, the two methods provide roughly equivalent rankings (Spearman rank correlation  = 0.97, Figure 3c). High-ranking populations are similar in both cases.

We note that SH and HED calculations on a network consider a taxon's distance from *all other* taxa. Thus, although the three northern populations are closely related to each other, they still receive high SH and HED scores because of the long branches separating them from the central and southern populations (Table 2; Figure 3c).

## Discussion

The premise of conservation below the species level is not novel. Faith's original [6] discussion of prioritizing taxa also considered populations on a tree. Several economically-important taxa have received population-level management since the late 1980s, *e.g.*, Atlantic salmon (*Salmo salar* Linnaeus 1758) [64], brown trout (*Salmo trutta* Linnaeus 1758) [65] and yellowfin tuna (*Thunnus albacares* Bonnaterre 1788) [66]. Managing species at the population level implies at least an informal ranking scheme, one which would rely, for example, on estimates of habitat patch size or effective population size [67]. Habitat degradation, climate change, and the demands of a growing human population have ensured the continued fragmentation of species' ranges over the next century (see, *e.g.*, pikas (*Ochotona princeps* Richardson 1828) [68]). In the midst of such rapid change, managing an imperiled species over its entire range may no longer be feasible, such that population rankings may be necessary.

Phylogenetic diversity measures have previously been adapted for non-treelike population genetic data (*e.g.*, [24], [25], [36]). However, the PD complementarity scores that can be obtained from these methods are contingent, *i.e.*, subject to change if extinction alters the shape of the network. Ours is the first ranking scheme to consider a taxon's contribution to all possible future networks (*sensu* Weitzman [23]), a potentially relevant framework for preserving future biodiversity. Given the stochastic nature of extinction, the general ranking systems offered by SH and HED may be more useful to wildlife managers than those that only consider the present structure of a phylogenetic network. Unlike previous approaches based on PD (*e.g.*, [24], [25], [36]), SH and HED rankings allow one to lengthen or shorten the list of taxa to conserve in the event that resources become more or less available.

Molecular techniques are now inexpensive and robust enough to make population genetic sampling a standard component of conservation planning, and we argue that a phylogenetic network approach offers insight into a species' population structure complementary to the current statistical assessments of differentiation employed by MUs and DPSs [16], [17]. We encourage researchers to employ such networks in future population genetic studies to provide conservation agencies with more informative analysis of datasets. Genotyping at multiple loci will provide more accurate estimates of population differentiation and allow for more sophisticated analyses of conservation-relevant processes such as recent demographic history and gene flow [69].

We acknowledge that the mathematical shortcomings of Φ_ST_ and *F*_ST_ estimators [70] may influence the magnitude and ranking of SH and HED scores, depending on the number of loci measured and the distribution of genetic diversity in a set of taxa. Our intention here is not to solve these theoretical problems but to demonstrate our network-based prioritization method with existing data. Newly-developed metrics such as Jost's *D* can be used to calculate SH and HED just as readily as traditional Φ_ST_ and *F*_ST_ distances, and we encourage the use of such unbiased estimators whenever such data are available. Indeed, any conservation metric of difference (*e.g.*, ecological, genomic, adaptability) can be used.

Several properties of the networks described here invite further investigation. In both our heuristic datasets, geographically peripheral populations are more genetically isolated, meaning they would rank highly on SH and HED. However, this was based on only very few putatively neutral markers. Two related questions concern how processes such as demographic history and current patterns of gene flow map onto genetic isolation as we measure it here, and also how phylogenetic networks map onto networks produced from ecological data (*e.g.*, niche use differences among populations).

We do not advocate relying solely on genetic isolation when deciding where and how resources should be allocated at the population level. Total population genetic diversity (*i.e.*, number of haplotypes) might also be considered. We note that in our examples, low-ranking populations tend to be geographically close to one another, meaning that their haplotypes are often shared. Important differences in ecology and adaptability [71] and current and future connectivity [72], must also be considered. However, our network approach and ranking system based on genetic differentiation can supplement existing systems of MUs and DPSs to improve the conservation of evolutionarily distinct populations in a world of increasing pressures and limited resources.

## Supporting Information

File S1 **Mathematical treatment of SH and HED and annotated R code for calculating both metrics.**
(PDF)

Table S1 Pairwise genetic distances (Φ_ST_) for spotted owl (*Strix occidentalis*) populations based on data from Barrowclough *et al*. [48], [51], with negative values set to zero.
(PDF)
