# Supplementary material for: Prioritizing Populations for Conservation Using Phylogenetic Networks
Source: PLoS One. 2014 Feb 28;9(2):e88945. doi: 10.1371/journal.pone.0088945 (PMC3938429; doi:10.1371/journal.pone.0088945)
Supplement: File S1 — Mathematical treatment of SH and HED and annotated R code for calculating both metrics. (PDF) [file pone.0088945.s001.pdf]

Supplementary material for: Prioritizing populations for conservation using phylogenetic networks

Logan Volkmann Iain Martyn Vincent Moulton
Andreas Spillner Arne Mooers

August 29, 2013

1 Preliminaries

Let X be a finite set of taxa and Σ a set of bipartitions or *splits* of X. Such a set Σ is called a *split system* on X. A split $S = \{A, B\}$ of X is denoted by $S = A|B$. For each split S of X and any $x \in X$ we denote by $S(x)$ the set in S that contains x and by $\overline{S}(x)$ the other set in S. A split system Σ is called *compatible* if its elements correspond precisely to the edges of some (necessarily unique) phylogenetic tree (cf. [7]). A *weighted* split system on X is a pair (Σ, λ) consisting of a split system Σ on X together with a map $\lambda : \Sigma \to \mathbb{R}_{\geq 0}$ that assigns a non-negative weight to each split in Σ.

The *phylogenetic diversity* of a subset $Y \subseteq X$ [8] relative to a weighted split system (Σ, λ) is given by:

$$PD_{(\Sigma,\lambda)}(Y) = \sum_{\substack{A|B\in\Sigma \\ A\cap Y\neq\emptyset, B\cap Y\neq\emptyset}} \lambda(A|B).$$

This quantity is also known as the *split diversity* [6]. It was originally defined for compatible split systems, i.e. for phylogenetic trees (see e.g. [9]). In particular, the phylogenetic diversity with respect to some tree is just the split diversity relative to the compatible split system corresponding to the tree, where split weights correspond to branch lengths.

1.1 The Shapley value (SH)

The Shapley value of a taxon x with respect to a phylogenetic tree on X, as defined in [3], can be extended to any weighted split system (Σ, λ) on X as follows:

$$\psi_x^{sh}(\Sigma, \lambda) = \frac{1}{|X|!} \sum_{Z\subseteq X, x\in Z} (|Z|-1)!(|X|-|Z|)!\big(PD_{(\Sigma,\lambda)}(Z) - PD_{(\Sigma,\lambda)}(Z - \{x\})\big).$$

Shapley values of elements relative to a tree correspond to values in this last formula relative to the corresponding compatible split system. As with phylogenetic trees, it is not hard to show that the Shapley value can also be written as the following linear combination of the weights of the splits in Σ:

$$\psi_x^{sh}(\Sigma, \lambda) = \sum_{S \in \Sigma} \frac{|\overline{S}(x)|}{|X||S(x)|} \lambda(S). \tag{1}$$

Note that, since the coefficients in this linear combination can be computed in polynomial time, the Shapley value of x with respect to (Σ, λ) can also be computed in polynomial time.

In case the split system Σ has a special structure, there can be efficient algorithms for computing the Shapley values $\psi_x^{sh}(\Sigma, \lambda)$ for all $x \in X$. For example, in case Σ is compatible there is an algorithm with run time in $O(n)$, $n = |X|$ [5]. Here we show that for so-called circular split systems there is an algorithm with run time in $O(n+m)$, $m = |\Sigma|$, for simultaneously computing Shapley values for all x in X.

A split system Σ is *circular* if there exists an ordering $x_1, x_2, \dots, x_n$ of the taxa in X such that for every split $A|B \in \Sigma$ there are $1 \leq i \leq j < n$ with $A = \{x_i, x_{i+1}, \dots, x_j\}$ or $B = \{x_i, x_{i+1}, \dots, x_j\}$. We shall say that such an ordering $x_1, x_2, \dots, x_n$ is *suitable* for the circular split system Σ. Note that split systems corresponding to NeighborNet networks are always circular [1] (a fact that also implies that NeighborNet networks can always be drawn in the plane without crossing edges).

Now, assume that we are given an ordering $x_1, x_2, \dots, x_n$ of the taxa in X that is suitable for Σ. Moreover, assume that, for each split $A|B \in \Sigma$, the two indices $1 \leq i \leq j < n$ with $\{x_i, x_{i+1}, \dots, x_j\} = A$ or $\{x_i, x_{i+1}, \dots, x_j\} = B$ are stored. Then, for any given $S \in \Sigma$ and any given $x \in X$, one can compute $|S(x)|$ in constant time and, therefore, by evaluating (1), we obtain $\psi_{x_1}^{sh}(\Sigma, \lambda)$ in $O(m)$ time.

Next, we perform a preprocessing of the splits in Σ. More specifically, we form subsets Σ_k, $1 \leq k < n$, by putting each split of the form $S = \{x_i, x_{i+1}, \dots, x_j\}|X - \{x_i, x_{i+1}, \dots, x_j\} \in \Sigma$ into Σ_j and also, in case $i > 1$, into Σ_{i-1}. Intuitively, the splits in Σ_k are those whose associated interval $\{x_i, x_{i+1}, \dots, x_j\}$ starts directly after taxon x_k or ends with taxon x_k.

Our algorithm for computing Shapley values processes the taxa $x_2, x_3, \dots, x_n$ in the given ordering. Assuming that the Shapley value $\psi_{x_{k-1}}^{sh}(\Sigma, \lambda)$ has already been computed, we now explain how to compute $\psi_{x_k}^{sh}(\Sigma, \lambda)$, $2 \leq k \leq n$. It is not hard to see that only the splits in Σ_{k-1} might change their contribution to Shapley values when going from x_{k-1} to x_k. So, first we subtract the values $\frac{|\overline{S}(x_{k-1})|}{|X||S(x_{k-1})|}\lambda(S)$ from $\psi_{x_{k-1}}^{sh}(\Sigma, \lambda)$, and then we add $\frac{|\overline{S}(x_k)|}{|X||S(x_k)|}\lambda(S)$ for each $S \in \Sigma_{k-1}$. This yields $\psi_{x_k}^{sh}(\Sigma, \lambda)$.

The $O(n+m)$ bound on the run time for this algorithm follows by observing that $\psi_{x_k}^{sh}(\Sigma, \lambda)$ can be computed in $O(|\Sigma_{k-1}|)$ time and noting that every split in Σ is contained in at most two of the sets Σ_h, $1 \leq h < n$.

Remark 1.1 To achieve the $O(n+m)$ run time the circular split system must be stored in a succinct way such as the one described above. Note that this is not the case for commonly used file formats, such as, for example, the *nexus*-format employed in the SplitsTree software package [4], which uses $O(n \cdot m)$ space to store an arbitrary split system Σ with m splits by listing, for each split in $S \in \Sigma$, the elements in one of the sets in S.

1.2 Heightened evolutionary distinctiveness (HED)

The HED value of a taxon x in X is computed in terms of probabilities $p(y)$ that are given for each element y in X. As with Shapley values, HED was originally defined for trees [10] but extends naturally to weighted split systems (Σ, λ) on X:

$$\psi_x^{hed}(\Sigma,\lambda) = \sum_{S\in\Sigma} \big(\prod_{y\in(S(x)-\{x\})} p(y)\big) \cdot \big(1 - \prod_{y\in\overline{S}(x)} p(y)\big) \cdot \lambda(S). \tag{2}$$

Again, HED values of elements relative to a tree correspond to values in this last formula relative to the corresponding compatible split system. The approach taken for computing the Shapley values for a weighted circular split system outlined above can be adapted for HED as follows. Note that for the special case of phylogenetic trees this was already shown in [5]. Here we briefly outline how $\psi_x^{hed}(\Sigma,\lambda)$ can be computed for all $x \in X$ in $O(n+m)$ time for any weighted circular split system (Σ, λ).

Let $x_1, x_2, \dots, x_n$ be an ordering of the taxa in X that is suitable for Σ. As for trees [5], we first perform a preprocessing step to compute, for every split $A|B \in \Sigma$, the values $\prod_{x\in A} p(x)$ and $\prod_{x\in B} p(x)$. To do this, we first perform a range minimum query preprocessing in $O(n)$ time so that afterwards we can decide in $O(1)$ time whether a given interval $x_i, x_{i+1}, \dots, x_j$ contains an element y with $p(y) = 0$ using a range minimum query [2]. This yields all the values $\prod_{x\in A} p(x)$ and $\prod_{x\in B} p(x)$, respectively, that are equal to 0 in $O(n+m)$ time. The remaining values can also be computed in $O(n+m)$ time by restricting the ordering $x_1, x_2, \dots, x_n$ to those $y \in X$ with $p(y) > 0$ and then computing the product over every prefix of the restricted ordering in $O(n)$ time. From these prefix-products the values $\prod_{x\in A} p(x)$ and $\prod_{x\in B} p(x)$, respectively, that are not equal to 0 can then be computed in constant time each, yielding $O(n+m)$ time in total, as claimed.

After finishing the preprocessing, we just use the algorithm for computing the Shapley values to compute $p(x)\psi_x^{hed}(\Sigma,\lambda)$ for all $x \in X$. This yields $\psi_x^{hed}(\Sigma,\lambda)$ for all $x \in X$ with $p(x) > 0$. For those $x \in X$ with $p(x) = 0$ we run a second phase just as we did for phylogenetic trees (cf. [5]).

References

[1] D. Bryant and V. Moulton. NeighborNet: an agglomerative method for the construction of phylogenetic networks. *Molecular Biology and Evolution*,

21:255–265, 2004.

[2] J. Fischer and V. Heun. A new succinct representation of RMQ-information and improvements in the enhanced suffix array. In *Proc. International Symposium on Combinatorics, Algorithms, Probabilistic and Experimental Methodologies*, volume 4614 of *LNCS*, pages 459–470. Springer, 2007.

[3] C.J. Haake, A. Kashiwada, and F.E. Su. The Shapley value of phylogenetic trees. *Journal of Mathematical Biology*, 56:479–497, 2008.

[4] D.H. Huson and D. Bryant. Application of phylogenetic networks in evolutionary studies. *Molecular Biology and Evolution*, 23:254–267, 2006.

[5] I. Martyn, T. Kuhn, A. Mooers, V. Moulton, and A. Spillner. Computing evolutionary distinctiveness indices in large scale analysis. *Algorithms for Molecular Biology*, 7, 2012.

[6] B. Minh, S. Klaere, and A. von Haeseler. Taxon selection under split diversity. *Systematic Biology*, 57:586–594, 2009.

[7] C. Semple and M. Steel. *Phylogenetics*. Cambridge University Press, 2003.

[8] A. Spillner, B. T. Nguyen, and V. Moulton. Computing phylogenetic diversity for split systems. *IEEE/ACM Transactions on Computational Biology and Bioinformatics*, 5:235–244, 2008.

[9] M. Steel. Phylogenetic diversity and the greedy algorithm. *Systematic Biology*, 54:527–529, 2005.

[10] M. Steel, A. Mimoto, and A. Mooers. Hedging our bets: the expected contribution of species to future phylogenetic diversity. *Evolutionary Bioinformatics*, 3:237–244, 2007.

```
#Scripts used for: "Prioritizing populations for conservation using phylogenetic
networks"
#L. Volkmann, I. Martyn, V. Moulton, A. Spillner, and A.Ø. Mooers

#Scripts prepared by I. Martyn, 2011
#Annotations by L. Volkmann, 2014

#Last Updated January 2014

#--------------------------------------------------------------------------
#-CONTENTS-----------------------------------------------------------------
#--------------------------------------------------------------------------

#-1: Calculating SH on a Phylogenetic Network------------------------------

#-2: Calculating HED on a Phylogenetic Network-----------------------------

#--------------------------------------------------------------------------

#Open the following libraries:

library(gdata)
library(motmot)

#-1------------------------------------------------------------------------
#-CALCULATING SH ON A PHYLOGENETIC NETWORK---------------------------------
#--------------------------------------------------------------------------

#This section generates Shapley (SH) rankings from a phylogenetic network (made in
SplitsTree).
#Load the NetworksSH function first (in a separate R script), then load the data file,
which contains output from SplitsTree.

file="PATH NAME"
file

#--------------------------------------------------------------------------
#NetworksSH function:

NetworksSH<-function(file)
{

#Read in the complete Nexus file but keep only the splits information:

CompleteNexus=scan(file, what = "", sep = "\n", quiet = TRUE,skip = 0)

#Identify beginning of splits block and allocate taxa:

st=grep("CYCLE",CompleteNexus)
taxset=unlist(strsplit(strsplit(CompleteNexus[st],"CYCLE")[[1]][2]," "))
taxset=unlist(strsplit(strsplit(strsplit(CompleteNexus[st],"CYCLE")[[1]][2],";")[[1]][
1]," "))
taxset=as.numeric(taxset[taxset>0])
taxset=taxset[!is.na(taxset)]

#Identify end of splits block:

edn=grep("END",CompleteNexus)
edn=edn[which(((edn-st)>0))[1]]
SplitsNexus=CompleteNexus[(st+2):(edn-2)]

#Identify all taxa names:
```

```
st=grep("TAXLABELS",CompleteNexus)
edn=grep("END",CompleteNexus)
edn=edn[which(((edn-st)>0))[1]]
NamesNexus=CompleteNexus[(st+1):(edn-2)]

#Verify that the split system is circular:

Names=matrix(0,length(NamesNexus),2)
for (i in 1:length(NamesNexus))
{number=strsplit(strsplit(strsplit(NamesNexus[i],"
")[[1]][1],"\\[")[[1]][2],"\\]")[[1]][1]
name=strsplit(NamesNexus[i]," ")[[1]][2]
name=strsplit(name,"'")[[1]][2]
Names[i,1]=name
Names[i,2]=number
}
TaxaNotInCycle=setdiff(as.numeric(Names[,2]),taxset)
if (!length(TaxaNotInCycle)==0)
{taxset=c(taxset,TaxaNotInCycle)
}

#Transform data from the splits block to manipulable, array form:

SplitsNexus=strsplit(SplitsNexus,"\t")
SplitsArray=matrix(0,length(SplitsNexus),3)
M=matrix(0,length(SplitsNexus),length(taxset))
for (i in 1:length(SplitsNexus))
{tmp=SplitsNexus[[i]]
sa1=strsplit(tmp[1],"[=]")[[1]][2]
sa1=strsplit(sa1,"]")[[1]][1]
sa2=as.numeric(tmp[2])
sa3=unlist(strsplit(tmp[3],"[ ,]"))
sa3=as.numeric(sa3[sa3>0])
SplitsArray[i,1]=as.numeric(sa1)
SplitsArray[i,2]=sa2
tmp2=intersect(taxset,sa3)
SplitsArray[i,3]=length(tmp2)
M[i,tmp2]=1
}

#Compute Shapley values.
#First taxa:

SH=as.vector(matrix(0,1,length(taxset)))
n=length(SH)
for (i in 1:(dim(M)[1]))
{
if (M[i,taxset[1]]==1)
{SH[1]=SH[1]+((n-SplitsArray[i,3])/(SplitsArray[i,3])*SplitsArray[i,2])
} else {
SH[1]=SH[1]+(SplitsArray[i,3]/(n-SplitsArray[i,3])*SplitsArray[i,2])
}
}

#All other taxa:

for (i in 2:n)
{SH[i]=SH[i-1]
enter=which((M[,taxset[(i-1)]]-M[,taxset[i]])==1)
leave=which((M[,taxset[(i-1)]]-M[,taxset[i]])==-1)
if (length(enter)==0)
```

```
{
} else {
for(j in 1:length(enter))
{a1=SplitsArray[enter[j],3]/(n-SplitsArray[enter[j],3])*SplitsArray[enter[j],2]
s1=((n-SplitsArray[enter[j],3])/(SplitsArray[enter[j],3])*SplitsArray[enter[j],2])
SH[i]=SH[i]+a1-s1
}
}
if (length(leave)==0)
{
} else {
for(k in 1:length(leave))
{a1=SplitsArray[leave[k],3]/(n-SplitsArray[leave[k],3])*SplitsArray[leave[k],2]
s1=((n-SplitsArray[leave[k],3])/(SplitsArray[leave[k],3])*SplitsArray[leave[k],2])
SH[i]=SH[i]-a1+s1
}
}
}

#Normalize and post-process:

SH=SH/length(taxset)

SH=as.matrix(SH[order(taxset)])
rownames(SH)=Names[,1]

tmp1=names(SH[order(SH,decreasing=TRUE),])
SH=matrix(SH[order(SH,decreasing=TRUE)],n,1)
rownames(SH)=tmp1

SH
}

#---------------------------------------------------------------------------
#Run the script on the data file:

NetworksSH(file)

#-2-------------------------------------------------------------------------
#-CALCULATING HED ON A PHYLOGENETIC NETWORK--------------------------------
#---------------------------------------------------------------------------

#This section generates Heightened Evolutionary Distinctiveness (HED) rankings from a
phylogenetic network (made in SplitsTree).
#Load the NetworksHED function first (in a separate R script), then load the data
file, which contains output from Splitstree.

file="PATH NAME"
file

#You will also need to load data file containing the probability of extinction for
each taxon.

ProbExtinctionFile="PATH NAME"
ProbExtinctionFile

#---------------------------------------------------------------------------
#NetworksHED function:

NetworksHED<-function(file,ProbExtinctionFile)
{
```

```
#Read in the complete Nexus file but keep only the splits information:

CompleteNexus=scan(file, what = "", sep = "\n", quiet = TRUE,skip = 0)

#Identify beginning of splits block and allocate taxa:

st=grep("CYCLE",CompleteNexus)
taxset=unlist(strsplit(strsplit(CompleteNexus[st],"CYCLE")[[1]][2]," "))
taxset=unlist(strsplit(strsplit(strsplit(CompleteNexus[st],"CYCLE")[[1]][2],";")[[1]][
1]," "))
taxset=as.numeric(taxset[taxset>0])
taxset=taxset[!is.na(taxset)]

#Identify end of splits block:

edn=grep("END",CompleteNexus)
edn=edn[which(((edn-st)>0))[1]]
SplitsNexus=CompleteNexus[(st+2):(edn-2)]

#Identify all taxa names:

st=grep("TAXLABELS",CompleteNexus)
edn=grep("END",CompleteNexus)
edn=edn[which(((edn-st)>0))[1]]
NamesNexus=CompleteNexus[(st+1):(edn-2)]

#Verify that the split system is circular:

Names=matrix(0,length(NamesNexus),2)
for (i in 1:length(NamesNexus))
{number=strsplit(strsplit(strsplit(NamesNexus[i],"
")[[1]][1],"\\[")[[1]][2],"\\]")[[1]][1]
name=strsplit(NamesNexus[i]," ")[[1]][2]
name=strsplit(name,"'")[[1]][2]
Names[i,1]=name
Names[i,2]=number
}
TaxaNotInCycle=setdiff(as.numeric(Names[,2]),taxset)
if (!length(TaxaNotInCycle)==0)
{taxset=c(taxset,TaxaNotInCycle)
}

#Transform data from the splits block to manipulable, array form:

SplitsNexus=strsplit(SplitsNexus,"\t")
SplitsArray=matrix(0,length(SplitsNexus),3)
M=matrix(0,length(SplitsNexus),length(taxset))
for (i in 1:length(SplitsNexus))
{tmp=SplitsNexus[[i]]
sa1=strsplit(tmp[1],"[=]")[[1]][2]
sa1=strsplit(sa1,"]")[[1]][1]
sa2=as.numeric(tmp[2])
sa3=unlist(strsplit(tmp[3],"[ ,]"))
sa3=as.numeric(sa3[sa3>0])
SplitsArray[i,1]=as.numeric(sa1)
SplitsArray[i,2]=sa2
tmp2=intersect(taxset,sa3)
SplitsArray[i,3]=length(tmp2)
M[i,tmp2]=1
}

#Read in and allocate probability of extinction values:
```

```
Probext=read.table(ProbExtinctionFile)
HED=as.vector(matrix(0,1,length(taxset)))
n=length(HED)
p=rep(0,n)
for (i in 1:n)
{tmp=Names[i,1]
p[i]=as.numeric(Probext[grep(tmp,Probext[,1]),2])
}

#Compute HED values.
#The first taxa:

HED=as.vector(matrix(0,1,length(taxset)))
for (i in 1:(dim(M)[1]))
{
if (M[i,taxset[1]]==1)
{tmp=which(M[i,]==1)
S=prod(p[tmp])/p[taxset[1]]
S_=prod(p[-tmp])
HED[1]=HED[1]+S*(1-S_)*SplitsArray[i,2]
} else {
tmp=which(M[i,]==1)
S_=prod(p[tmp])
S=prod(p[-tmp])/p[taxset[1]]
HED[1]=HED[1]+S*(1-S_)*SplitsArray[i,2]
}
}

#All other taxa:

for (i in 2:n)
{factmult=(p[taxset[(i-1)]]/p[taxset[i]])
HED[i]=HED[(i-1)]*factmult
enter=which((M[,taxset[(i-1)]]-M[,taxset[i]])==1)
leave=which((M[,taxset[(i-1)]]-M[,taxset[i]])==-1)

if (length(enter)==0)
{
} else {
for(j in 1:length(enter))
{tmp=which(M[enter[j],]==1)
S=prod(p[-tmp])/p[taxset[i]]
S_=prod(p[tmp])
a=S*(1-S_)*SplitsArray[enter[j],2]
S_=prod(p[-tmp])
S=prod(p[tmp])/p[taxset[(i-1)]]
b=S*(1-S_)*SplitsArray[enter[j],2]
HED[i]=HED[i]+a-b*factmult
}
}

if (length(leave)==0)
{
} else {
for(k in 1:length(leave))
{tmp=which(M[leave[k],]==1)
S=prod(p[-tmp])/p[taxset[(i-1)]]
S_=prod(p[tmp])
a=S*(1-S_)*SplitsArray[leave[k],2]
S_=prod(p[-tmp])
S=prod(p[tmp])/p[taxset[i]]
```

```
b=S*(1-S_)*SplitsArray[leave[k],2]
HED[i]=HED[i]+b-a*factmult
}
}
}

#Normalize and post-process:

HED=HED/length(taxset)

HED=as.matrix(HED[order(taxset)])
rownames(HED)=Names[,1]

tmp1=names(HED[order(HED,decreasing=TRUE),])
HED=matrix(HED[order(HED,decreasing=TRUE)],n,1)
rownames(HED)=tmp1

HED
}

#---------------------------------------------------------------------------
#Run the script on the data file:
```
