# Supplementary material for: Prioritizing Populations for Conservation Using Phylogenetic Networks
Source: PLoS One. 2014 Feb 28;9(2):e88945. doi: 10.1371/journal.pone.0088945 (PMC3938429; doi:10.1371/journal.pone.0088945)
Supplement: Table S1 — Pairwise genetic distances (ΦST) for spotted owl (Strix occidentalis) populations based on data from Barrowclough et al. [48], [51], with negative values set to zero. (PDF) [file pone.0088945.s002.pdf]

Table S1. Pairwise genetic distances (Φ_{ST}) for spotted owl (*Strix occidentalis*) populations based on data from Barrowclough *et al.* [48,51], with negative values revised to zero. See Table 1 for an explanation of abbreviations used.

	Zion	Reef	Mant	Cann	Flag	Coco	Mogo	SanF	Tula	Pino	Blac	Sacr	Cata	Rinc	Rita	Huac
Zion	—															
Reef	0	—														
Mant	0.5496	0.6018	—													
Cann	0.1869	0.2808	0	—												
Flag	0.0503	0.1529	0.0737	0	—											
Coco	0.4154	0.4511	0.0027	0.0118	0.0109	—										
Mogo	0.2280	0.2851	0.0557	0	0	0	—									
SanF	0.3219	0.3821	0.0509	0	0	0.0498	0.0041	—								
Tula	0.3453	0.3658	0.0122	0	0	0	0	0	—							
Pino	0.3460	0.3919	0.0597	0	0	0	0	0	0	—						
Blac	0.4880	0.5250	0.0792	0.0186	0.1237	0.0292	0	0.0966	0	0.0529	—					
Sacr	0.4622	0.4995	0.1921	0.0309	0.1870	0.2264	0.1721	0.1077	0.1656	0.0707	0.2152	—				
Cata	0.3697	0.4436	0	0	0	0	0	0	0	0	0	0.1594	—			
Rinc	0.6521	0.6769	0.1817	0.2033	0.3044	0.0203	0.1387	0.1687	0.0346	0.1419	0.0629	0.3069	0.0262	—		
Rita	0.3145	0.3684	0.0137	0	0	0.0618	0	0	0	0	0.0333	0	0	0.2006	—	
Huac	0.6929	0.7193	0.5522	0.2705	0.4368	0.2273	0.2468	0.3099	0.1880	0.3750	0.1862	0.3233	0.2529	0.4382	0.1873	—
Pina	0.6171	0.6540	0.2113	0.0500	0.2222	0	0.0295	0.0829	0	0.0687	0	0.2233	0	0	0.0877	0.4764
Zaca	0.5591	0.6298	0	0	0	0	0	0	0	0.0455	0	0.0496	0	0.4000	0	0.6000
Agua	0.5367	0.6047	0.4286	0	0.1429	0.3697	0.1830	0.1159	0.2214	0.2222	0.3885	0.0091	0.3125	0.7273	0	0.8788
Olym	0.8192	0.8320	0.8127	0.7510	0.7826	0.8108	0.7711	0.7738	0.7737	0.7966	0.7990	0.7749	0.8021	0.8565	0.7648	0.8588
Wena	0.8426	0.8531	0.8486	0.7809	0.8128	0.8269	0.7935	0.7981	0.7901	0.8257	0.8221	0.7969	0.8301	0.8805	0.7935	0.8850
Will	0.7966	0.8080	0.7807	0.7263	0.7572	0.7819	0.7453	0.7474	0.7466	0.7657	0.7673	0.7468	0.7713	0.8176	0.7395	0.8217
Shas	0.7532	0.7670	0.7203	0.6728	0.7042	0.7454	0.7012	0.7007	0.7096	0.7126	0.7217	0.7016	0.7197	0.7689	0.6865	0.7700
Humb	0.7755	0.7828	0.7600	0.7203	0.7423	0.7646	0.7356	0.7365	0.7366	0.7448	0.7500	0.7374	0.7524	0.7830	0.7303	0.7880
Mari	0.8570	0.8670	0.8734	0.7848	0.8265	0.8279	0.7956	0.8014	0.7872	0.8412	0.8283	0.7972	0.8433	0.8995	0.8012	0.9121
Lass	0.5061	0.5309	0.5104	0.3973	0.4153	0.5495	0.4553	0.4577	0.4902	0.4523	0.5394	0.5095	0.4906	0.6145	0.4127	0.6196
ElDo	0.7634	0.7680	0.8615	0.7089	0.7387	0.7278	0.6955	0.7118	0.6691	0.7633	0.7775	0.7382	0.7832	0.8626	0.7237	0.8913
Sequ	0.7829	0.7859	0.8864	0.7360	0.7659	0.7451	0.7168	0.7355	0.6874	0.7909	0.7971	0.7585	0.8078	0.8824	0.7509	0.9115
Carm	0.8205	0.8220	0.9611	0.7590	0.8122	0.7528	0.7273	0.7492	0.6864	0.8388	0.8169	0.7650	0.8494	0.9322	0.7799	0.9765
SanB	0.8601	0.8576	0.9738	0.8198	0.8614	0.7892	0.7803	0.8016	0.7297	0.8792	0.8552	0.8122	0.8875	0.9476	0.8363	0.9830
SanJ	0.8601	0.8576	0.9738	0.8198	0.8614	0.7892	0.7803	0.8016	0.7297	0.8792	0.8552	0.8122	0.8875	0.9476	0.8363	0.9830
Palo	0.7972	0.8019	0.9517	0.7208	0.7808	0.7341	0.6976	0.7191	0.6643	0.8137	0.7950	0.7384	0.8257	0.9231	0.7443	0.9724

Pina	Zaca	Agua	Olym	Wena	Will	Shas	Humb	Mari	Lass	ElDo	Sequ	Carm	SanB	SanJ	Palo
—															
0.5000	—														
0.8182	1	—													
0.8406	0.7965	0.8020	—												
0.8719	0.8395	0.8454	0	—											
0.8015	0.7589	0.7663	0.1332	0.0732	—										
0.7450	0.6919	0.6939	0.1546	0.1199	0.0279	—									
0.7705	0.7359	0.7431	0.1323	0.0493	0	0.0091	—								
0.8990	0.8811	0.8796	0.2966	0.2795	0.1383	0.1300	0.1186	—							
0.5609	0.4611	0.4252	0.6739	0.6936	0.6511	0.6063	0.6463	0.6942	—						
0.8727	0.8844	0.8730	0.8749	0.8934	0.8421	0.7991	0.7983	0.9115	0.0661	—					
0.8967	0.9098	0.9011	0.8852	0.9035	0.8523	0.8098	0.8070	0.9225	0.0970	0.0209	—				
0.9732	1	1	0.8933	0.9165	0.8549	0.8056	0.8042	0.9449	0.0857	0.0742	0.0019	—			
0.9810	1	1	0.9148	0.9337	0.8781	0.8345	0.8237	0.9576	0.1381	0.1169	0.0357	0	—		
0.9810	1	1	0.9148	0.9337	0.8781	0.8345	0.8237	0.9576	0.1381	0.1169	0.0357	0	0	—	
0.9680	1	1	0.8812	0.9069	0.8427	0.7908	0.7948	0.9374	0.0578	0.0507	0	0	0	0	—
